# BthTX-II, an Asp49 PLA_2_ from *Bothrops jararacussu*, Impairs *Toxoplasma gondii* Infection: In Vitro and Ex Vivo Approaches

**DOI:** 10.3390/ph18091260

**Published:** 2025-08-25

**Authors:** Vinícius Queiroz Oliveira, Emanuelle Lorrayne Ferreira, Lorena Pinheiro Morais, Leonardo Alves Garcia, Gabriel de Oliveira Sousa, Marcos Paulo Oliveira Almeida, Guilherme de Souza, Joed Pires de Lima Júnior, Natália Carine Lima dos Santos, Rafael Martins de Oliveira, Tássia Rafaela Costa, Andreimar Martins Soares, Luísa Carregosa Santos, Daiana Silva Lopes, Emidio Beraldo-Neto, Angelica Oliveira Gomes, Jovita Eugênia Gazzinelli Cruz Madeira, Bellisa Freitas Barbosa, Eloisa Amália Vieira Ferro, Samuel Cota Teixeira, Veridiana de Melo Rodrigues Ávila

**Affiliations:** 1 Laboratory of Biochemistry and Animal Toxins, Institute of Biotechnology, Universidade Federal de Uberlândia, Uberlândia 38405-318, MG, Brazil; viniciusqueirozbio@gmail.com (V.Q.O.); emanuelle.ferreira@ufu.br (E.L.F.); lorena10morais@gmail.com (L.P.M.); garcia7557alves@gmail.com (L.A.G.); gabrieldeosousa@ufu.br (G.d.O.S.); 2 Laboratory of Immunophysiology of Reproduction, Institute of Biomedical Sciences, Universidade Federal de Uberlândia, Uberlândia 38405-318, MG, Brazil; marcospaulooliveiraalmeida@hotmail.com (M.P.O.A.); guisbio@hotmail.com (G.d.S.); joedjunior07@gmail.com (J.P.d.L.J.); carinenatalia@yahoo.com.br (N.C.L.d.S.); rafael_martinso@hotmail.com (R.M.d.O.); bellisafb@ufu.br (B.F.B.); eloisa.ferro@ufu.br (E.A.V.F.); 3 Laboratory of Biotechnology of Proteins and Bioactive Compounds in the Western Amazon (LABIOPROT), Fundação Oswaldo Cruz (FIOCRUZ Rondônia), Universidade Federal de Rondônia (UNIR), National Institute of Science and Technology of Epidemiology of the Western Amazon, INCT-EPIAMO, Porto Velho 76812-245, RO, Brazil; tassiarcosta@yahoo.com.br (T.R.C.); andreimarsoares@gmail.com (A.M.S.); 4 Institute Multidisciplinary in Health, Universidade Federal da Bahia, Vitória da Conquista 45029-094, BA, Brazil; luisacarregosabio@gmail.com (L.C.S.); lsdaiana@yahoo.com.br (D.S.L.); 5 Laboratory of Biochemistry, Instituto Butantan, São Paulo 05503-900, SP, Brazil; emidio.beraldo@butantan.gov.br; 6 Laboratory of Cell Interactions, Institute of Natural and Biological Sciences, Universidade Federal do Triângulo Mineiro, Uberaba 38025-350, MG, Brazil; angelica.gomes@ufu.br; 7 Laboratory of Micotoxins, Fundação Ezequiel Dias, Belo Horizonte 30510-010, MG, Brazil; jovigazzinelli@gmail.com

**Keywords:** congenital toxoplasmosis, phospholipase A_2_, trophoblast, snake venom

## Abstract

**Background/Objectives**: *Toxoplasma gondii*, an obligate intracellular parasite, poses a major global health concern owing to its potential for congenital transmission, particularly during pregnancy. Current pharmacological treatments, including spiramycin and pyrimethamine, exhibit limitations in both efficacy and safety, underscoring the need for novel therapeutic strategies. In this study, we investigated the antiparasitic potential of BthTX-II, an Asp49 phospholipase A_2_ (PLA_2_) isolated from *Bothrops jararacussu* venom, in human trophoblast cells (BeWo) and third-trimester human placental explants infected with *T. gondii*. **Methods**: In vitro assays were performed using BeWo cells infected with *T. gondii* tachyzoites and treated with non-cytotoxic concentrations of BthTX-II (3.125, 1.56, and 0.78 µg/mL). An ex vivo model employing third-trimester human placental villous explants was used under similar conditions. Parasite proliferation, adhesion, and invasion were assessed alongside host immune response modulation. **Results**: Our findings demonstrate that BthTX-II reduces *T. gondii* proliferation in BeWo cells at all tested non-cytotoxic concentrations. The toxin also significantly impaired parasite adhesion and invasion while modulating host immune response by upregulating interleukin (IL)-6, IL-8, and macrophage migration inhibitory factor (MIF), and downregulating vascular endothelial growth factor—potentially disrupting parasite proliferation. In placental villous explants, BthTX-II (1.56 μg/mL) reduced *T. gondii* proliferation and modulated IL-8, MIF, and tumour necrosis factor-alpha levels without compromising tissue viability. **Conclusions**: These findings highlight BthTX-II as a potential candidate in toxoplasmosis treatment. Further investigation should focus on its dual role in limiting parasite development and modulating immune responses at the maternal–fetal interface.

## 1. Introduction

*Toxoplasma gondii* is an obligate intracellular protozoan parasite of the phylum Apicomplexa and the causative agent of toxoplasmosis, a widespread zoonotic disease affecting both animals and humans [1,2]. Its seroprevalence is notably high in tropical regions of South America, as well as in parts of Europe and Asia [3]. In particular, congenital toxoplasmosis has a global incidence of approximately 1.5 cases per 1000 live births, with the risk of maternal infection increasing as pregnancy progresses [4,5]. Brazil is a geographically large country with considerable socioeconomic and healthcare disparities. Between 2019 and 2024, more than 26,000 cases of congenital toxoplasmosis were reported in the country [6].

In pregnant women, infection may occur during or shortly before pregnancy, allowing tachyzoites to cross the placenta and infect the foetus. This can lead to severe complications, including stillbirth, prematurity, growth restriction, neurological and ocular impairments, and miscarriage [1,7,8]. Early maternal diagnosis is crucial for preventing congenital toxoplasmosis and enables timely treatment to reduce the risk of foetal transmission [9].

Maternal immune adaptations are essential for sustaining pregnancy, as cells at the maternal–foetal interface establish a tolerogenic microenvironment within the placenta that supports foetal development [10]. Therefore, a delicate balance of antagonistic immunological processes is required during gestational *T. gondii* infection. Cytokines are key regulators that play a central role in orchestrating the immune response and influencing various pathophysiological processes that contribute to disease progression. Both pro- and anti-inflammatory cytokines are critical for initiating and maintaining innate and adaptive immunity to limit *T. gondii* proliferation [11,12]. Cytokine expression undergoes dynamic modulation across different stages of pregnancy. During early gestation, elevated concentrations of interleukin (IL)-4, IL-10, IL-8, and CCL2 indicate a Th2-dominant immune profile. In mid-pregnancy, increased levels of IFN-γ, tumour necrosis factor-alpha (TNF-α), IL-1β, and IL-6 indicate a mixed Th1/Th2 response. At parturition, IL-4, IL-10, IL-8, and CCL2 concentrations increase again, while those of IFN-γ, TNF-α, IL-1β, and IL-6 decline, shifting the immune environment back toward a Th2-biased profile [13].

Treatment of infected pregnant individuals often begins with spiramycin to prevent transmission to the foetus; however, its limited ability to cross the placenta restricts its effectiveness against foetal infections [14,15]. Consequently, pyrimethamine and sulfadiazine, combined with folinic acid, are used as first-line treatment when foetal infection is confirmed [14]. However, this standard treatment can cause significant side effects and toxicity, including bone marrow suppression and teratogenic effects [14]. Additionally, conventional therapies have been associated with the development of drug-resistant parasites [16,17]. Despite the availability of alternative therapies, such as atovaquone and azithromycin, a significant gap remains in comprehensive clinical data regarding the tolerability and potential adverse effects [18,19]. These limitations underscore the urgent need to identify more selective, safer, and more effective therapeutic strategies.

From this perspective, animal toxins have gained attention due to their therapeutic potential, as they comprise a diverse collection of biomolecules capable of effectively interfering with various physiological mechanisms. Among the molecules already known to mediate physiological events are the bradykinin-potentiating peptides (BPPs), isolated from the venom of *Bothrops jararaca*, which served as the basis for the development of the antihypertensive agent Captopril^®^ [20]. Additionally, the binding of disintegrins to the integrin αIIbβ3 prevents fibrinogen from interacting with the receptor, thereby inhibiting platelet aggregation. Two current drugs, Tirofiban (Aggrastat^®^) and Eptifibatide (Integrilin^®^), were developed based on disintegrins like echistatin isolated from the venom of *Ecchis carinatus* and barbourin isolated from the venom of the *Sistrurus miliarius barbourin*, respectively [21,22].

In this context, animal-derived toxins have attracted considerable interest for their antiparasitic properties, especially in relation to *Toxoplasma gondii* [23,24]. Among these, phospholipases A_2_ (PLA_2_s) from snake venom are the most prominent proteins known for their anti-parasitic properties [25,26,27].

PLA_2_s are enzymes widely distributed among living organisms that hydrolyse the 2-acyl ester bond of membrane phospholipids, releasing fatty acids and lysophosphatides [28,29]. PLA_2_s derived from Viperidae snake venom (svPLA_2_s) are categorised into two main groups: (i) catalytically active PLA_2_s, which possess a calcium-binding site coordinated by the β-carboxylic group of the Asp49 residue, as well as the carbonyl groups of Tyr28, Gly30, and Gly32, and two structurally conserved water molecules that form the coordination sphere for Ca^2+^ binding [30,31,32]; and (ii) catalytically inactive PLA_2_s, in which the substitution of Asp49 (negatively charged side chain) with Lys49 (positively charged side chain) significantly alters the Ca^2+^ binding site, preventing ion coordination and resulting in minimal or no catalytic activity [33,34].

The anti-*T*. *gondii* effect induced by svPLA_2_s was first demonstrated by previous work of our research group [25]. We showed that BnSP-7, a Lys49 PLA_2_ from *Bothrops pauloensis* venom, inhibited *T. gondii* growth at a lower dose than that required to induce cytotoxicity in HeLa cells and also modulated the host immune response. Recently, our group reported the prominent antiparasitic effect of MjTX-II, another Lys49 PLA_2_ from *Bothrops moojeni*, in congenital toxoplasmosis models. We demonstrated that MjTX-II impairs parasite infection by targeting both the reactive oxygen species and vascular endothelial growth factor (VEGF) pathways [27]. In this context, the potential anti-*T. gondii* effects of catalytically active svPLA_2_s remain unexplored. Therefore, to gain insight into the mechanisms by which svPLA_2_s act against *T. gondii* infection, we investigated the antiparasitic effect of BthTX-II using an in vitro model with human trophoblast cells and an ex vivo model using human placental villous explants from the third trimester of pregnancy.

BthTX-II, an Asp49 PLA_2_, is a key component of *Bothrops jararacussu* venom. It shares a high degree of homology with other snake venom Asp49 PLA_2_s and contains critical residues—Tyr28, Gly30, Gly32, and particularly Asp49—that form the Ca^2+^-binding loop, along with His48, Tyr52, and Asp99, all of which are directly or indirectly involved in catalysis [35,36]. BthTX-II possesses a distorted Ca^2+^-binding loop and a modification in the C-terminal region that results in an alternative dimeric quaternary structure [37,38]. This structural modification contributes to its low PL activity [38]. Nevertheless, BthTX-II retains significant biological effects, including the induction of oedema and myotoxicity [39], participation in inflammatory responses [40,41,42], and the exhibition of antitumour, antimetastatic, and antiangiogenic properties [43,44]. Considering these promising effects induced by BthTX-II, we report here for the first time the impact of an Asp49 PLA_2_ on the regulation of *T. gondii* parasitism in the context of congenital toxoplasmosis, using both in vitro and ex vivo approaches.

## 2. Results

### 2.1. BthTX-II Inhibits Proliferation of T. gondii Without Causing Toxicity to Trophoblasts

**Figure 1:**
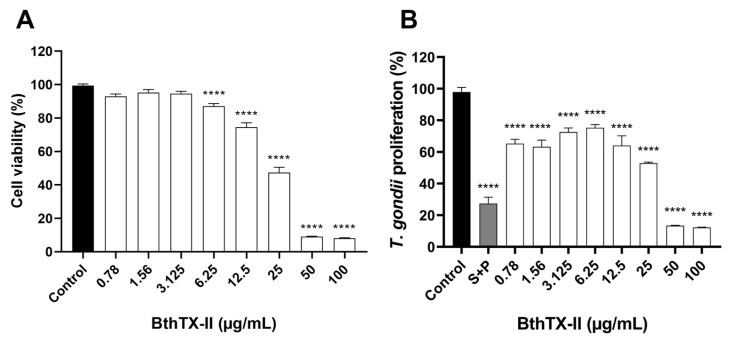
Toxin derived from *Bothrops jararacussu* venom (BthTX-II) affects human trophoblast cell (BeWo) viability and inhibits *Toxoplasma gondii* proliferation. (**A**) BeWo cells were treated for 24 h in twofold serial dilutions of BthTX-II (ranging from 100 to 0.78 μg/mL) or with culture medium alone (control group). Cell viability was expressed as a percentage. Cells in the control group were considered to have 100% viability. (**B**) *T. gondii*-infected BeWo cells were treated for 24 h with BthTX-II at the same concentration range, a combination of sulfadiazine and pyrimethamine (S+P; 200 µg/mL + 8 µg/mL, respectively), or culture medium alone (control group, considered as supporting 100% parasite proliferation). Asterisks (*) indicate a statistically significant difference compared to the control group. **** *p* < 0.0001.

Cell viability was initially assessed using the [(3-(4,5-dimethylthiazol-2-yl)-2,5-diphenyltetrazolium bromide)] (MTT) assay to determine safe concentrations of BthTX-II for subsequent experiments. BeWo cells were exposed to a twofold serial dilution of BthTX-II (ranging from 100 to 0.78 μg/mL) for 24 h. As shown in Figure 1A, concentrations above 6.25 μg/mL were cytotoxic to trophoblasts compared to the control group (**** *p* < 0.0001), with a half-maximal cytotoxic concentration of 17.53 ± 1.09 μg/mL.

To evaluate the half-maximal inhibitory concentration (IC_50_) of BthTX-II on *T. gondii*, BeWo cells were infected and treated with BthTX-II (ranging from 100 to 0.78 μg/mL) for 24 h. All concentrations significantly inhibited *T. gondii* proliferation compared to the control group (**** *p* < 0.0001) (Figure 1B). As expected, sulfadiazine and pyrimethamine (S+P) treatment significantly reduced *T. gondii* proliferation compared to the untreated group (**** *p* < 0.0001) (Figure 1B). BthTX-II exhibited an IC_50_ value of 20.1 ± 1.13 μg/mL. Based on these findings, the three lowest non-cytotoxic concentrations of BthTX-II (3.125, 1.56, and 0.78 μg/mL), which also inhibited parasite proliferation, were selected for subsequent antiparasitic assays.

### 2.2. BthTX-II Directly Affects Parasites and Impairs Early Stages of T. gondii Infection in BeWo Cells, but Its Antiparasitic Effect Is Lost After Treatment Removal

**Figure 2:**
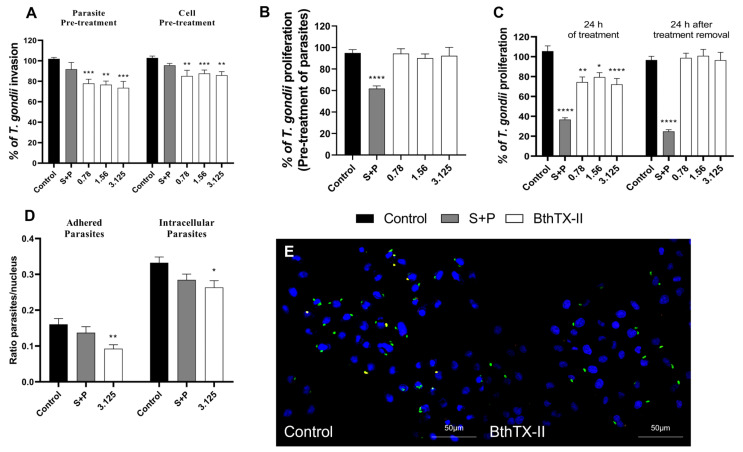
BthTX-II directly interferes with early steps of *T. gondii* infection. (**A**) BeWo cells (incubated for 24 h) and *T. gondii* tachyzoites (incubated for 1 h) were treated with BthTX-II (3.125, 1.56, or 0.78 μg/mL). After treatment, parasites were allowed to adhere for 3 h to previously fixed BeWo cells, and the percentage of adhered parasites (% of *T. gondii* adhesion) was measured using β-galactosidase activity. In both experimental conditions, intracellular parasite proliferation was quantified using the β-galactosidase assay. (**B**) *T. gondii* tachyzoites (1 × 10^6^) were incubated with BthTX-II (3.125, 1.56, or 0.78 μg/mL), S+P, or culture medium alone for 1 h, followed by co-incubation with BeWo cells (3 × 10^4^ cells/well in 200 μL) in 96-well microplates for 24 h. Parasite intracellular proliferation was assessed using a colorimetry-based β-galactosidase assay and expressed as a percentage relative to the control (% of *T. gondii* proliferation). (**C**) Infected BeWo cells were treated with BthTX-II (3.125, 1.56, or 0.78 μg/mL), S+P, or culture medium alone for 24 h. In parallel, treatments were removed from infected cells in duplicate wells, and cells were maintained in treatment-free medium for an additional 24 h. (**D**) BeWo cells (1 × 10^5^/well in 500 μL) were cultured in 24-well microplates containing 13 mm coverslips and incubated for 18 h at 37 °C and 5% CO_2_. *T. gondii* tachyzoites were pre-incubated with BthTX-II (3.125 μg/mL) or culture medium (untreated parasites) for 30 min prior to host cell interaction. Following a 3 h invasion period, intracellular parasites (green+/red− fluorescence) and adhered parasites (red fluorescence or co-localised red+/green+ signals [yellow]) were quantified across 20 randomly selected fields using fluorescence microscopy. Nuclei were stained with TO-PRO-3 iodide (blue). (**E**) Representative fluorescence images show the effects of BthTX-II (3.125 μg/mL) on tachyzoite–host cell interactions, including intracellular parasites (green+/red−), adhered parasites (red or co-localised red+/green+ [yellow]), and host cell nuclei (blue). Scale bar: 50 μm. Asterisks (*) indicate statistically significant differences compared to the control group. * *p* < 0.05; ** *p* < 0.01; *** *p* < 0.001; **** *p* < 0.0001.

To investigate the potential of BthTX-II to disrupt the initial stages of *T. gondii* infection, we investigated whether pre-treatment of *T. gondii* tachyzoites (1 h) or BeWo cells (24 h) with BthTX-II (3.125, 1.56, and 0.78 μg/mL) could selectively affect the parasites or host cells, respectively. The selected concentrations of BthTX-II significantly reduced parasite invasion by approximately 20% following pre-treatment of the parasite (** *p* < 0.01; *** *p* < 0.001) (Figure 2A). In contrast, S+P treatment did not significantly alter invasion compared to the untreated (control) group. Similarly, pre-treatment of BeWo cells reduced invasion by approximately 10% (** *p* < 0.01; *** *p* < 0.001) (Figure 2A). Additionally, S+P treatment showed no significant effect on the early stages of parasite infection compared to the untreated group.

To validate these findings, we performed a red/green assay to distinguish between adhered and intracellular parasites. Pre-treatment of BeWo cells with BthTX-II (3.125 μg/mL) for 24 h significantly decreased the number of adhered (** *p* < 0.01) and intracellular (* *p* < 0.05) parasites compared to the control group (Figure 2D). Additionally, S+P treatment did not alter the early stages of infection compared to the untreated control group (Figure 2D). Representative fluorescence microscopy images show a higher number of adhered (red/yellow) and intracellular (green) parasites in the control group compared to that in the BthTX-II-treated (3.125 μg/mL) group (Figure 2E). Taken together, our findings suggest that BthTX-II significantly disrupts the early stages of *T. gondii* infection.

Additionally, we pre-treated *T. gondii* with BthTX-II (3.125, 1.56, and 0.78 μg/mL) for 1 h to evaluate whether the protein affects the parasite’s proliferative capacity. Notably, no significant differences were observed in the ability to inhibit parasitic proliferation before infection (Figure 2B). Furthermore, we investigated whether the toxin could maintain its antiparasitic effect after treatment discontinuation. BthTX-II effectively controlled *T. gondii* proliferation (**** *p* < 0.0001); however, proliferation was restored once BthTX-II was removed (Figure 2C).

### 2.3. BthTX-II Upregulates the IL-6, IL-8, and Macrophage Migration Inhibitory Factor (MIF) Levels and Downregulates VEGF Production in T. gondii-Infected BeWo Cells

To investigate the effect of BthTX-II (3.125 μg/mL) in modulating the host immune response triggered by BeWo cells during *T. gondii* infection, we measured the cytokines IL-4, IL-6, IL-8, IL-10, and MIF.

**Figure 3:**
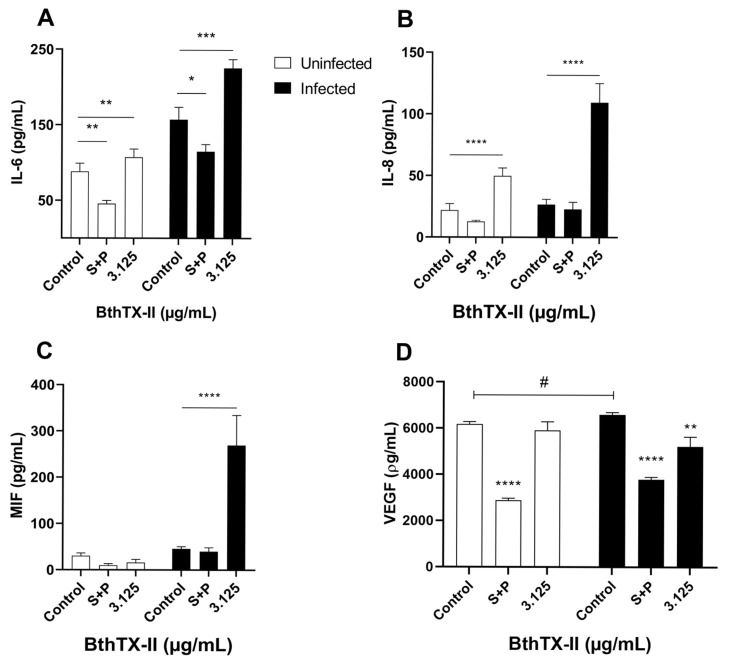
BthTX-II upregulates interleukin (IL)-6, IL-8, and migration inhibitory factor (MIF) production in cells and downregulates vascular endothelial growth factor (VEGF) levels in T. gondii-infected BeWo. BeWo cells, uninfected or infected with *T. gondii* tachyzoites (multiplicity of infection of 3) for 3 h, were treated for 24 h with BthTX-II (3.125 μg/mL) or culture medium alone. Cell culture supernatants were collected for measurement of (**A**) IL-6, (**B**) IL-8, (**C**) MIF, and (**D**) VEGF levels. Asterisks (*) indicate a statistically significant difference compared to the control group. * *p* < 0.05; ** *p* < 0.01; *** *p* < 0.001; **** *p* < 0.0001. The hash symbol (#) indicates a statistically significant difference between control groups.

BthTX-II (3.125 μg/mL) treatment upregulated IL-6 levels in both uninfected (** *p* < 0.01) and infected (*** *p* < 0.001) cells compared to those in the uninfected, untreated and infected, untreated cells, respectively (Figure 3A). In contrast, S+P treatment reduced IL-6 production in both uninfected (* *p* < 0.05) and infected (** *p* < 0.01) cells compared to that in the uninfected, untreated and infected, untreated cells, respectively (Figure 3A).

Similarly, BthTX-II (3.125 μg/mL) led to a marked increase in IL-8 levels in both uninfected and infected cells compared to those in the uninfected, untreated and infected, untreated cells (**** *p* < 0.0001) (Figure 3B). Conversely, no significant differences in IL-8 levels were observed between the control and S+P groups under infected or non-infected conditions (Figure 3B).

Moreover, BthTX-II treatment significantly increased MIF production only in *T. gondii*-infected BeWo cells (**** *p* < 0.0001) compared to that in the infected, untreated cells (Figure 3C). In uninfected groups, neither BthTX-II nor S+P treatments modulated MIF levels. Similarly, S+P treatment did not affect MIF production in infected BeWo cells compared to that in the infected, untreated cells (Figure 3C).

The effect of BthTX-II on VEGF production by BeWo cells was also investigated. *T. gondii* infection alone upregulated VEGF levels compared to those in uninfected, untreated BeWo cells. (# *p* < 0.05). BthTX-II (3.125 μg/mL) significantly reduced VEGF levels in infected BeWo cells compared to that in uninfected, untreated and infected, untreated cells (** *p* < 0.01; **** *p* < 0.0001) (Figure 3D). In contrast, the standard treatment with S+P did not significantly alter VEGF levels in *T. gondii*-infected BeWo cells compared to those in the untreated control cells (Figure 3D).

IL-4 and IL-10 were undetectable in supernatants from BeWo cells.

### 2.4. BthTX-II Reduces T. gondii Proliferation in Human Placental Explants

To further validate the findings in BeWo cells, we assessed the impact of BthTX-II on the susceptibility of placental explants to *T. gondii* infection.

**Figure 4:**
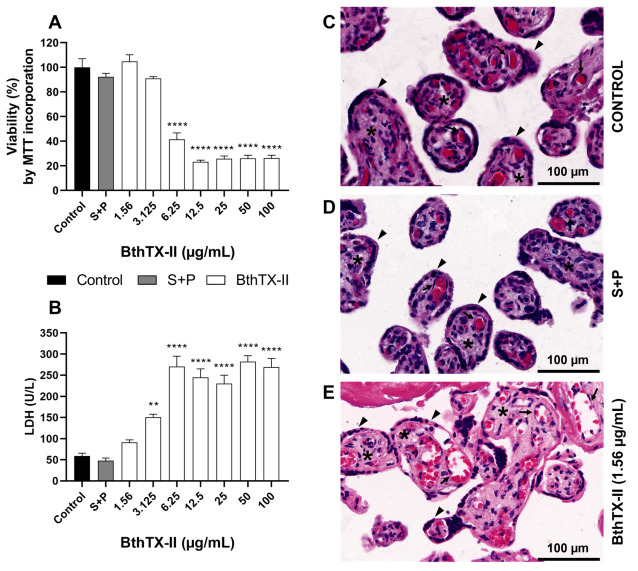
Viability of human placental villous explants after treatment with BthTX-II. Villous explants were incubated for 24 h with BthTX-II (ranging from 100 to 1.56 μg/mL) or culture medium and subjected to viability analysis. (**A**) MTT assays were performed on villous explants, and tissue viability is shown as a percentage of MTT incorporation. (**B**) Supernatants from villous explants were collected to measure lactate dehydrogenase (LDH) (U/L) levels. (**C**–**E**) Haematoxylin–eosin (HE)-stained histological sections were evaluated. Representative fields, at 20× magnification with a 100 µm scale bar, demonstrate tissue morphology of (**C**) untreated (control), (**D**) S+P-treated, or (**E**) BthTX-II-treated (1.56 µg/mL) placental explants. Arrowheads indicate the intact multinucleated syncytiotrophoblast outer layer; asterisks denote the densely cellular mesenchymal tissue covered externally by the syncytiotrophoblast and internally filling the placental explant structure; arrows indicate healthy foetal blood vessels, surrounded by mesenchyme, composed of intact endothelial cells, and containing conserved circulating foetal blood cells, mostly eosinophilic foetal erythrocytes. Asterisks (*) indicate a significant difference compared to the control group. ** *p* < 0.01; **** *p* < 0.0001.

Cell viability was initially evaluated using the MTT assay, which revealed significant cytotoxicity at concentrations of 6.25 μg/mL and above, compared to that in the control group (**** *p* < 0.0001) (Figure 4A). Similarly, in the lactate dehydrogenase (LDH) assay, BthTX-II was toxic to the villous tissue at 1.56 μg/mL (** *p* < 0.01) and from 6.25 to 100 μg/mL (**** *p* < 0.0001) compared to the untreated group (Figure 4B). To validate these biochemical findings, histological sections stained with haematoxylin–eosin (HE) were analysed to assess tissue morphology following BthTX-II treatment. The explants treated with BthTX-II (1.56 μg/mL) displayed morphology comparable to the untreated group, preserving syncytiotrophoblast structural integrity and the presence of intact red blood cells within the vessels (Figure 4C–E). As expected, S+P treatment did not affect tissue viability (Figure 4A,B,D).

**Figure 5:**
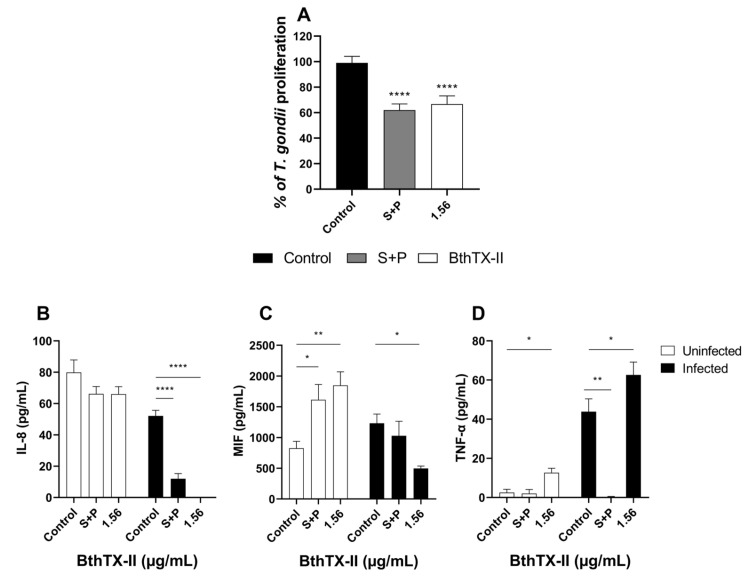
BthTX-II inhibits *T. gondii* proliferation in human placental villous explants and modulates IL-8, MIF, and tumour necrosis factor-alpha (TNF-α) cytokine production. Villous explants were incubated for 24 h with BthTX-II (3.125, 1.56, and 0.78 μg/mL), S+P (150 µg/mL + 200 µg/mL, respectively), or culture medium alone. (**A**) Villous explants infected with *T. gondii* tachyzoites (1 × 10^6^ parasites/villous/well) were treated with BthTX-II (3.125, 1.56, and 0.78 μg/mL), S+P (150 + 200 μg/mL, respectively), or culture medium alone for 24 h, and *T. gondii* intracellular proliferation was measured using a β-galactosidase assay. Supernatants from uninfected and infected villous explants after treatments were collected to quantify IL-8 (**B**), MIF (**C**), and TNF-α (**D**). Cytokine levels were expressed in pg/mg of tissue. Data are presented as mean ± standard error of the mean. Asterisks (*) indicate statistically significant differences compared to the control group. * *p* < 0.05; ** *p* < 0.01; **** *p* < 0.0001.

Corroborating our in vitro findings, treatment with 1.56 μg/mL BthTX-II significantly reduced parasite proliferation compared to that in the control group (*** *p* < 0.001, Figure 5A). Additionally, S+P treatment also effectively controlled the infection (**** *p* < 0.0001, Figure 5A).

### 2.5. BthTX-II Modulates IL-8, MIF, and TNF-α Production in T. gondii-Infected Human Placental Explants

To evaluate the immunomodulatory effects of BthTX-II, we measured the secretion of cytokines (IL-4, IL-6, IL-8, IL-10, TNF-α, and MIF) from human villous explants under various experimental conditions.

Our results demonstrated that both BthTX-II (1.56 μg/mL) and S+P treatments significantly reduced IL-8 production in *T. gondii*-infected placental explants compared to that in the infected, untreated explants (**** *p* < 0.0001; Figure 5B). In contrast, IL-8 levels remained unchanged in uninfected explants treated with BthTX-II compared with those in the untreated control group.

Treatment with BthTX-II (1.56 μg/mL) (* *p* < 0.05) and S+P (** *p* < 0.01) significantly upregulated MIF in uninfected explants compared to that in the uninfected, untreated explants (Figure 5C). In contrast, upon *T. gondii* infection, BthTX-II (1.56 μg/mL) treatment significantly reduced MIF levels compared to those in the infected, untreated explants (* *p* < 0.05), while no significant difference was observed between the S+P-treated and untreated control groups (Figure 5C).

BthTX-II (1.56 μg/mL) treatment significantly increased TNF-α production (* *p* < 0.05) in uninfected explants compared to that in the uninfected, untreated control explants. Conversely, no significant difference was observed between the S+P-treated and uninfected, untreated groups (Figure 5D). In *T. gondii*-infected explants, S+P treatment downregulated TNF-α levels compared to those in the infected, untreated explants (** *p* < 0.01; Figure 5D).

IL-4, IL-6, and IL-10 production were undetectable in supernatants from BeWo cells.

## 3. Discussion

Congenital toxoplasmosis remains a significant global health challenge. Lack of an effective vaccine or therapeutic treatment underscores the urgent need to identify and develop alternative treatments that are both safe for mothers and foetuses and capable of preventing the vertical transmission of *T. gondii* [7,45]. To address this need, several studies have explored compounds from diverse sources for their potential inhibitory effects and mechanisms of action against the parasite. Among these, snake venom has garnered attention owing to its anti-protozoal properties against infectious diseases [26] such as malaria [46], trypanosomiasis, leishmaniasis [47], and toxoplasmosis [27]. In this study, we investigated the effects of BthTX-II, a catalytically active PLA_2_ from *B. jararacussu*, on *T. gondii* using in vitro and ex vivo models of the maternal–foetal interface.

BthTX-II effectively inhibited *T. gondii* proliferation at significantly lower concentrations than the other PLA_2_s. For instance, Borges et al. (2016) reported that BnSP-7, a Lys49 PLA_2_ from *B. pauloensis* venom, required concentrations of 50, 25, and 12.5 μg/mL to effectively suppress *T. gondii* proliferation in infected HeLa cells [25]. Similarly, Teixeira et al. (2025) evaluated the Lys49 PLA_2_ MjTX-II from *B. moojeni* venom in *T. gondii*-infected BeWo cells and observed that concentrations ranging from 10 to 40 μg/mL reduced *T. gondii* proliferation [27]. Our findings reveal that BthTX-II inhibits *T. gondii* proliferation at markedly lower doses without inducing cytotoxicity in host cells, highlighting its enhanced potency as a candidate for therapeutic intervention. These differences among various PLA_2_s are expected, as even minor structural variations can significantly influence their biological effects.

These findings are consistent with those of other studies that investigated Asp49 PLA_2_s against various pathogens. For example, Cassani et al. (2023) showed that BthTX-II impairs both the early and post-entry stages of the ZIKV replicative cycle [48]. Similarly, Cecilio et al. (2013) demonstrated that BlD-PLA_2_, an Asp49 from *Bothrops leucurus*, exhibits antiviral activity against the dengue virus [49]. Sudarshan and Dhananjaya (2016) reported that NN-XIb-PLA_2_, an acidic Asp49 from *Naja naja*, exerts antibacterial effects against several microbial species [50]. Similarly, the VRV-PL-VIIIa from *Daboia russelii pulchella* inhibited the growth of both Gram-positive and Gram-negative bacteria [51]. Additionally, Barros et al. (2016) found that liposomes containing BthTX-II exhibited in vitro anti-*Leishmania amazonensis* activity [52]. Collectively, these findings suggest that Asp49 PLA_2_s hold promising biotechnological potential and should be further explored as novel drug candidates.

Next, we evaluated whether the reduction in parasite proliferation was due to an effect of the toxin on the parasite, the host cell, or both. Our data indicate that BthTX-II pretreatment of both cells and parasites significantly impaired *T. gondii*’s invasive capacity in invasion assays, suggesting that the toxin alters host cell pathways critical for parasite entry and also directly affects the parasite. Teixeira et al. (2025) demonstrated that MjTX-II binds to the intercellular adhesion molecule 1, which plays a key role in parasite dissemination [27]. This interaction compromised both the early and late stages of the *T. gondii* lytic cycle. Therefore, the reduced invasion observed in pre-treated cells may be related to the binding of BthTX-II to host molecules involved in parasite entry. However, to date, no specific receptors for BthTX-II have been identified, and there is no direct evidence of its binding to host or parasite targets; therefore, further studies are required to test this hypothesis. Additionally, pretreatment with *T. gondii* tachyzoites did not affect their proliferative capacity, indicating that the inhibitory activity of BthTX-II does not exert a lasting effect on intracellular growth. This supports the notion that the primary mechanism of action of this toxin may be host cell-dependent. Notably, the antiparasitic effect of BthTX-II was reversed upon its removal, suggesting that the continuous presence of the toxin is required to sustain host-intrinsic mechanisms that control *T. gondii* proliferation.

Subsequently, we investigated which host mechanisms are triggered by BthTX-II that may contribute to the control of *T. gondii* proliferation. This effect appears to be closely associated with modulation of the host immune response, particularly through changes in cytokine and growth factor secretion. In infected BeWo trophoblast cells, BthTX-II treatment led to a marked increase in IL-6, IL-8, and MIF levels, suggesting a shift toward a pro-inflammatory environment conducive to parasite control. Cytokine modulation is closely associated with the ability of trophoblasts to control *T. gondii* infection [53,54,55]. These findings are consistent with those of previous studies, indicating that these cytokines play key roles in controlling infection within maternal–foetal interface models [53,54,55].

According to Jebbari et al. (1998), IL-6 plays a crucial role in both the inflammatory response and its resolution [56]. IL-6 is associated with several physiological processes, including embryo implantation, fertility, foetal development, and nutrient transfer [57,58]. During *T. gondii* infection, IL-6 is upregulated in response to the parasite. This elevation may enhance the host cell’s ability to mount an effective defence against *T. gondii*, as previous studies have shown that IL-6 plays a protective role during infection by promoting localised inflammation and maintenance of pregnancy [53,59].

MIF functions as a critical regulator of immune and inflammatory responses, serving as a key cytokine in the defence against pathogens, including *T. gondii* [11,60]. In the presence of MIF, macrophages infected with *T. gondii* produce pro-inflammatory cytokines characteristic of the M1 activation profile, whereas the absence of MIF results in a marked reduction in these cytokines [61]. The regulation of infection by MIF is also observed in placental explants, where increased levels of this cytokine during the first trimester, as well as its stimulation in the third trimester, contribute to a reduction in *T. gondii* DNA content [62,63]. IL-8 is a pro-inflammatory cytokine frequently associated with inflammation and acts as a key mediator in toxoplasmosis. Its expression is typically associated with *T. gondii* infection [64,65]. The increased release of chemokines, including IL-8, contributes to the recruitment of neutrophils to sites of infection, forming part of the inflammatory infiltrate commonly observed during acute or reactivated toxoplasmosis [66]. IL-8 acts as a potent neutrophil chemoattractant, and in vitro studies have demonstrated that human neutrophils can inhibit *T. gondii* replication, highlighting its role in controlling parasite proliferation during the early stages of infection [67,68]. Recent studies have shown that macrophage-derived MIF activates the extracellular signal-regulated kinase (ERK) signalling pathway in both murine and human systems, leading to the induction of IL-8 production [69,70]. This interaction suggests a potential synergistic role between MIF and IL-8 in shaping early immune responses.

The ability of BthTX-II to enhance the production of IL-6, IL-8, and MIF may play a key role in modulating the immune response and limiting *T. gondii* infection in BeWo cells. These findings suggest a potential immunomodulatory effect of BthTX-II, promoting a pro-inflammatory environment that aids in parasite control.

svPLA_2_s can specifically target various sites, including cell membranes and receptors, across different tissues [28,71]. Their pharmacological effects arise from their interactions with both phospholipids and proteins. One well-characterised membrane receptor for svPLA_2_s is the VEGF receptor 2 (VEGFR-2) [72]. A previous study demonstrated that a Lys49-PLA_2_ isolated from *Agkistrodon piscivorus* venom binds with high affinity to VEGFR-2 via its C-terminal loop region, leading to specific inhibition of endothelial cell growth [72]. Notably, *T. gondii* infection increases VEGF production, which activates the protein kinase B and ERK1/2 signalling pathways. This activation promotes a positive feedback loop that amplifies VEGF expression, creating a favourable environment for parasite growth and replication [73].

The present study demonstrates a significant decrease in VEGF levels in the supernatant of *T. gondii*-infected BeWo cells following treatment with BthTX-II. This reduction in VEGF secretion correlates with a marked inhibition of parasite proliferation, suggesting a potential mechanism involving the modulation of VEGF signalling pathways. By interfering with these pathways, known to support parasite survival and replication, BthTX-II may disrupt the cellular environment necessary for effective parasite growth. Consistent with our findings, Teixeira et al. (2025) reported reduced VEGF secretion following treatment with the Lys49 PLA_2_ MjTX-II in *T. gondii*-infected BeWo cells [27]. Furthermore, other studies have demonstrated that BnSP-7, MjTX-II, and BthTX-II downregulate VEGF production in various experimental models, reinforcing the association between svPLA activity and VEGF signalling pathways [44,74,75].

To support the in vitro findings and confirm the effectiveness of BthTX-II in inhibiting *T. gondii* infection, we employed third-trimester human villous explants as an ex vivo model. We verified that the non-cytotoxic concentration of BthTX-II (1.56 μg/mL)—as confirmed by viability assays, LDH assessments, and histological analysis using HE staining—also inhibited the intracellular proliferation of *T. gondii*.

Given the complexity of immune responses during pregnancy, we evaluated the impact of BthTX-II on cytokine production in villous explants. In contrast to BeWo cells, BthTX-II reduced the secretion of IL-8 and MIF in villi infected by *T. gondii* while upregulating TNF-α production. The production of proinflammatory cytokines, particularly TNF-α, plays a fundamental role in controlling parasite proliferation during the early stages of infection [76]. This is supported by studies showing that TNF-α neutralisation increases susceptibility and parasite burden and that elevated TNF-α levels have been reported in retinochoroiditis associated with congenital toxoplasmosis [77,78]. Although the immunological profiles were modulated differently, the antiparasitic mechanisms observed in the ex vivo model may involve additional pathways or molecules that contribute to parasite control. These differences in cytokine modulation between in vitro and ex vivo models are expected, as the ex vivo model contains not only villous trophoblasts but also mesenchymal tissue and blood vessels, which are absent in cell cultures [79]. Taken together, our results indicate that BthTX-II exerts its antiparasitic effects, at least in part, by modulating host immune responses in a context-dependent manner. By enhancing or redirecting cytokine and growth factor signalling, including IL-6, IL-8, MIF, VEGF, and TNF-α, BthTX-II promotes an immune environment that limits *T. gondii* replication and supports host defence at the maternal-foetal interface. These results position BthTX-II as a promising candidate for further investigation using various models of the maternal–foetal interface.

## 4. Materials and Methods

### 4.1. Preparation of Asp49-PLA_2_ BthTX-II

BthTX-II used in this study was obtained from the Toxin Repository of the Laboratory of Biochemistry and Animal Toxins (LABITOX), following the recommended purification and storage protocols [80].

### 4.2. PL Activity of BthTX-II

The PLA_2_ activity was confirmed as previously described [81]. Activity was measured using egg yolk as the substrate in the presence of 0.03 M sodium deoxycholate and 0.6 M CaCl_2_. The mixture was incubated with 5 µg of BthTX-II at room temperature, and the enzymatic activity was assessed via potentiometric titration. PLA_2_ activity was expressed in U/mg, where one unit of PL activity corresponds to one µEq NaOH/min/mg.

### 4.3. Cell Culture and Parasite Maintenance

Human trophoblast cells (BeWo lineage) were obtained from the American Type Culture Collection (CCL-98^TM^, Manassas, VA, USA) and cultured in Roswell Park Memorial Institute (RPMI) 1640 medium (Cultilab, Campinas, SP, Brazil) supplemented with 100 U/mL penicillin (Sigma Chemical Co., St. Louis, MO, USA), 100 μg/mL streptomycin (Sigma), and 5% foetal bovine serum (FBS) (Cultilab) [65].

To control for variability due to cell passage, all experiments were conducted using cells within a limited passage range. The cell passages are an important factor in cell culture, and in our studies, we limited them to 10 passages according to Liao et al., 2014 [82].

*T. gondii* tachyzoites (RH strain, clone 2F1), which constitutively express the β-galactosidase gene, were maintained through serial passages in BeWo cells cultured in RPMI 1640 medium supplemented with 2% FBS, 100 U/mL penicillin, and 100 μg/mL streptomycin [65]. All cell and parasite cultures were maintained at 37 °C in a humidified atmosphere containing 5% CO_2_.

### 4.4. Human Placental Explants

Third-trimester human placentas (36–40 weeks of gestation, N = 9) were collected following elective caesarean deliveries at the Hospital de Clínicas, Universidade Federal de Uberlândia, MG, Brazil. The study was approved by the appropriate Ethics Committee (approval number: 7.407.162). Placental tissues were selected based on predefined exclusion criteria. Individuals were excluded if they presented with conditions such as preeclampsia, chronic hypertension, active infections (e.g., toxoplasmosis, leishmaniasis, trypanosomiasis), chorioamnionitis, chronic kidney or heart disease, connective tissue disorders, pre-existing or gestational diabetes mellitus, or any other medical issues that could confound the study outcomes [65,83].

For the human villous explants, these were obtained exclusively from third-trimester placentas, and biological replicates were derived from multiple independent donors (n = 3). We ensured that all donors met the same inclusion criteria and followed standardised collection and processing protocols to minimise inter-donor variability. This approach was adopted to increase the reproducibility and translational relevance of our findings. Floating terminal chorionic villous explants (~10 mm^3^) were dissected and individually placed into 96-well microplates (one villous per well), each containing 200 μL of fresh RPMI 1640 medium supplemented with 100 U/mL penicillin, 100 μg/mL streptomycin, and 10% FBS. The cultures were incubated for 24 h at 37 °C and 5% CO_2_.

### 4.5. Cell Viability in BeWo Cells

BeWo cells (3 × 10^4^ cells/well in 100 µL) were cultured in 96-well plates for 24 h in RPMI 1640 medium supplemented with 10% FBS at 37 °C and 5% CO_2_. Following incubation, the cells were treated for 24 h with serial two-fold dilutions of BthTX-II (100, 50, 25, 12.5, 6.25, 3.125, 1.56, or 0.78 µg/mL) in RPMI medium or with medium alone as a control. Subsequently, cell viability was assessed via incubation with 10 µL of MTT (5 mg/mL) in 90 µL of RPMI medium containing 10% FBS for 4 h at 37 °C and 5% CO_2_. The formazan crystals were solubilised by adding a solution of 10% sodium dodecyl sulphate (SDS; Sigma) and 50% *N*,*N*-dimethylformamide (DMF; Sigma), followed by incubation for 30 min. Absorbance was measured at 570 nm using a microplate reader (Titertek Multiskan Plus, Flow Laboratories, McLean, VA, USA). Data are expressed as the percentage of viable cells relative to untreated control cells (100% cell viability). Dose-response inhibition curves using the log(inhibitor) vs. normalised response model with variable slope were generated as previously described [84]. Experiments were conducted in triplicate, each with eight technical replicates.

### 4.6. Proliferation of T. gondii

To evaluate the effects of BthTX-II on *T. gondii* intracellular proliferation, a β-galactosidase assay was performed. BeWo cells (3 × 10^4^/well in 100 µL) were seeded in 96-well microplates containing medium with 10% FBS at 37 °C and 5% CO_2_. The cells were then infected with *T. gondii* tachyzoites at a multiplicity of infection (MOI) of 3:1 (three parasites per cell). After 3 h, the medium was removed, extracellular parasites were eliminated by washing with 1× PBS, and cells were treated with BthTX-II (100, 50, 25, 12.5, 6.25, 3.125, 1.56, or 0.78 µg/mL) for 24 h at 37 °C and 5% CO_2_. Cells treated with S+P (200 µg/mL + 8 µg/mL, respectively) served as positive controls [65]. Untreated *T. gondii*-infected BeWo cells were used as negative controls. After 24 h of treatment, culture supernatants were collected and stored at −80 °C for subsequent cytokine level measurements. *T. gondii* intracellular proliferation was assessed using a colorimetric β-galactosidase assay. The number of tachyzoites was quantified relative to a standard curve generated with free 2F1 tachyzoites (1 × 10^6^ to 15,625 × 10^3^ parasites) [55,85].

### 4.7. Invasion and Proliferation Pre-Treating T. gondii or BeWo

To evaluate the effect of BthTX-II on *T. gondii* invasion and intracellular proliferation, three different pretreatment models were employed. In the first model, BeWo cells (3 × 10^4^/well in 100 µL) were seeded in 96-well microplates in 10% FBS medium at 37 °C and 5% CO_2_. *T. gondii* tachyzoites at an MOI of 3 (three parasites per cell) were pretreated with BthTX-II at non-toxic concentrations (3.125, 1.56, and 0.78 μg/mL), S+P (200 + 8 μg/mL), or maintained in culture medium alone (control group) for 1 h at 37 °C and 5% CO_2_. Cells were then infected with the pre-treated *T. gondii* tachyzoites. After 24 h of treatment, intracellular proliferation was assessed using a colorimetric β-galactosidase assay. The data were expressed as a percentage of *T. gondii* proliferation, with the number of parasites in BeWo cells infected with untreated parasites (negative control) considered 100%.

In the second model, BeWo cells (3 × 10^4^ cells/well) were seeded in 100 µL of RPMI medium supplemented with 10% FBS in 96-well culture plates. Subsequently, *T. gondii* tachyzoites at an MOI of 3 were pre-incubated for 1 h at 37 °C and 5% CO_2_ with non-toxic concentrations of BthTX-II (3.125, 1.56, and 0.78 μg/mL), S+P (200 + 8 μg/mL), or culture medium alone (control group). The parasites were then centrifuged and resuspended in 10% FBS medium, and the cells were infected for 3 h. Next, the cells were washed multiple times with serum-free RPMI medium to remove excess extracellular (non-internalised) parasites. Finally, *T. gondii* intracellular invasion was quantified using a colorimetric β-galactosidase assay.

In the third model, BeWo cells (3 × 10^4^ cells/well) were pretreated for 24 h at 37 °C and 5% CO_2_ with non-toxic concentrations of BthTX-II (3.125, 1.56, and 0.78 μg/mL), S+P (200 + 8 μg/mL), or culture medium alone (control group). After discarding the supernatant, the cells were washed with serum-free RPMI medium. The cells were then infected with *T. gondii* tachyzoites at an MOI of 3 for 3 h. Finally, *T. gondii* intracellular invasion was assessed using a colorimetric β-galactosidase assay. Results were expressed as a percentage of *T. gondii* invasion, with the number of parasites in infected, untreated BeWo cells (negative control) considered 100%. Experiments were performed in triplicate, each with eight technical replicates.

### 4.8. Reversibility Assay

The persistence of the antiparasitic effect of BthTX-II was assessed using a reversibility assay, as previously described [86,87]. Initially, BeWo cells (3 × 10^4^ cells/well in 100 μL) were seeded in 96-well microplates and infected with *T. gondii* tachyzoites at an MOI of 3 for 3 h at 37 °C and 5% CO_2_. Subsequently, non-internalised parasites were removed by washing with 1× PBS. The cells were incubated with BthTX-II (3.125, 1.56, and 0.78 μg/mL), S+P (200 + 8 μg/mL), or maintained in culture medium alone (control group) at 37 °C and 5% CO_2_. After 24 h, the supernatant was discarded, and intracellular parasite proliferation was quantified. In parallel, in a separate microplate, the treatment was removed after 24 h, and the infected cells were incubated in treatment-free medium for an additional 24 h before quantifying the number of tachyzoites. In both conditions, intracellular *T. gondii* proliferation was quantified using the β-galactosidase assay, as previously described. Finally, the reversibility rate was calculated as a percentage (treatment reversibility %) at 24 h post-treatment removal, comparing it to the untreated group (considered 100%) and the corresponding treatment conditions at 24 h (baseline for comparison). Experiments were performed in triplicate, each with eight technical replicates.

### 4.9. Adhesion and Invasion Assay (“Red/Green Assay”)

The green/red antibody differential staining assay was used to confirm the role of BthTX-II in the early stages of *T. gondii* infection, as previously described [65,83,88]. BeWo cells (1 × 10^5^/well in 500 μL) were cultured in 24-well microplates containing 13 mm coverslips and incubated for 18 h at 37 °C and 5% CO_2_. Next, *T. gondii* tachyzoites were added to microcentrifuge tubes in the presence of BthTX-II (3.125 μg/mL), S+P (200 + 8 μg/mL), or culture medium alone (untreated parasites) for 30 min at 37 °C and 5% CO_2_. The parasites were then centrifuged and resuspended in treatment-free medium. The cells were infected with the pre-treated *T. gondii* tachyzoites at an MOI of 3 for 3 h. The cells were then washed twice with 1× PBS to remove non-internalised parasites. To label adhered parasites, the cells were fixed with 4% paraformaldehyde for 15 min at room temperature, followed by incubation with a rabbit polyclonal anti-*T. gondii* primary antibody (#20530; Abcam, Waltham, MA, USA) diluted 1:500 in PGN (PBS containing 0.25% gelatin) for 1 h at room temperature. This was followed by incubation with Alexa Fluor 594-conjugated anti-rabbit IgG (#A11012; Invitrogen, Waltham, MA, USA) diluted 1:500 in PGN. To label the intracellular parasites, the coverslips were incubated for 1 h with the same primary antibody diluted 1:500 in PGN containing 0.01% saponin (permeabilisation solution), followed by incubation for 1 h with Alexa Fluor 488-conjugated anti-rabbit IgG (#A11008; Invitrogen, Waltham, MA, USA) and a nuclear marker, TO-PRO-3 Iodide (Life Technologies, Waltham, MA, USA), both diluted 1:500 in permeabilisation solution. The coverslips were mounted on glass slides and analysed using confocal fluorescence microscopy (40× objective, LSM 510 Meta, Zeiss, Oberkochen, Germany). The number of intracellular (green+/red−) and attached [red+ or green+/red+ (yellow)] parasites was counted in 20 randomly selected fields per coverslip [66]. We normalised the data by the ratio (number of parasites/cell nucleus) of each experimental condition. Experiments were performed in duplicate, each with four technical replicates.

### 4.10. Viability of Placental Villous Explants

Placental villous explants were collected and cultured for 24 h. Tissue viability was assessed using MTT, LDH, and histological assays according to established protocols [83]. In brief, villous explants were treated with serial two-fold dilutions of BthTX-II in RPMI medium (100, 50, 25, 12.5, 6.25, 3.125, 1.56, or 0.78 µg/mL) or culture medium alone for 24 h at 37 °C and 5% CO_2_. In addition, the combination of S+P (150 + 200 μg/mL, respectively) was used as a safe and effective dosage against *T. gondii* in this ex vivo model [65]. Supernatants from the explants were collected to measure LDH activity following the manufacturer’s instructions (LDH Liquiform, Labtest Diagnóstica S.A., Lagoa Santa, MG, Brazil; #86-2/30, Lot 7010). The LDH released into the culture medium was quantified in U/L and used as an indicator of tissue integrity.

In parallel, under the same experimental conditions, tissue viability was assessed using the MTT assay. Following treatment, culture supernatants were discarded and replaced with 200 µL of MTT solution (180 µL of culture medium plus 20 µL of MTT [5 mg/mL]) and incubated for 4 h at 37 °C and 5% CO_2_. Subsequently, the formazan crystals formed from MTT reduction were solubilised by adding 100 µL of a solution containing 10% SDS in water and DMF (1:1), followed by incubation for 18 h at 37 °C and 5% CO_2_. Next, villi were harvested from each well, and absorbance at 570 nm was measured using a microplate reader (Versa Max enzyme-linked immunosorbent assay [ELISA] Microplate Reader, Molecular Devices, Sunnyvale, CA, USA). Tissue viability was expressed as a percentage based on MTT incorporation, with the absorbance of villi incubated in culture medium alone (untreated group) considered 100% viable. As an additional approach to validate the tissue viability of the treated placental explants, morphological evaluation was performed. Formalin-fixed, paraffin-embedded sections of the explants were stained with HE, then digitalised using an Aperio VERSA 200 BF & Fluorescence scanner (Leica VERSA 200 BF & Fluorescence; Leica Biosystems, Wetzlar, Germany). Following morphological validation, representative fields were captured at 20× magnification with a 100 µm scale bar using the Aperio ImageScope software (version 12.4.6.5003; Leica Biosystems). Experiments were performed in duplicate, each with eight technical replicates.

### 4.11. T. gondii Infection in Human Placental Villous Explants

We evaluated the intracellular proliferation of *T. gondii* in placental explants using a colorimetry-based β-galactosidase assay. Placental explants were collected and seeded in 96-well microplates (one villous per well in 200 µL) containing supplemented culture medium for 24 h at 37 °C and 5% CO_2_. The villous explants were then infected with *T. gondii* tachyzoites (1 × 10^6^/well in 200 µL) for 24 h and subsequently treated with a nontoxic concentration of BthTX-II (1.56 μg/mL), S+P (150 + 200 μg/mL), or culture medium alone for another 24 h at 37 °C and 5% CO_2_. Placental villi were collected and stored at −80 °C for subsequent analyses, including protein quantification using the Bradford reagent and assessment of intracellular *T. gondii* proliferation via β-galactosidase activity [65]. Experiments were performed in duplicate, each with eight technical replicates.

### 4.12. Cytokine and VEGF Determination

Cytokine assays were performed to assess the host immune responses to *T. gondii* infection under different experimental conditions. The concentrations of human cytokines IL-4, IL-6, IL-8, IL-10, TNF-α, and MIF released into the culture supernatants of BeWo cells or placental villi were quantified using ELISA kits, following the manufacturers’ instructions (BD Biosciences, San Diego, CA, USA; R&D Systems, Minneapolis, MN, USA). Data are expressed in pg/mL, based on cytokine-specific standard curves specific for BeWo cell samples. For placental explants, cytokine levels were normalised by calculating the ratio of cytokine concentration (pg/mL) to the corresponding total protein content (μg/mL) of each sample, with results expressed as pg/mg of tissue. The detection limits for each cytokine were established from standard curves as follows: IL-4 (500 pg/mL), IL-6 (300 pg/mL), IL-8 (400 pg/mL), IL-10 (500 pg/mL), TNF-α (500 pg/mL), and MIF (6000 pg/mL).

VEGF levels in the supernatant of BeWo cells were measured using a commercial Cytokine Bead Array kit for cellular proteins (BD Biosciences, San Jose, CA, USA), following the manufacturer’s instructions. VEGF levels were calculated based on a standard curve with a detection limit of 10 pg/mL. Samples were analysed using a CytoFLEX flow cytometer (Beckman Coulter, Brea, CA, USA) and FlowJo software (version 7.6.3).

### 4.13. Statistical Analysis

All data are presented as mean ± standard error of the mean ± SEM. Data were tested for normality. If they met parametric criteria, they were analysed using one-way ANOVA followed by Sidak’s multiple comparison test. For non-parametric data, the Kruskal–Wallis test followed by Dunn’s multiple comparisons post-test was used. Statistical analyses were performed using GraphPad Prism^®^ version 8.0 (GraphPad Software, Inc., San Diego, CA, USA). Results were considered statistically significant at * *p* < 0.05, ** *p* < 0.01, *** *p* < 0.001, and **** *p* < 0.0001.

## 5. Conclusions

Our findings demonstrate that BthTX-II exhibits significant anti-*Toxoplasma gondii* activity by effectively inhibiting key stages of the parasite’s life cycle, including adhesion, invasion, and proliferation within trophoblast cells and placental explants. Furthermore, BthTX-II modulates the host immune response, which is critical for the parasite’s intracellular survival. The observed downregulation of VEGF suggests a potential interaction with the VEGFR-2, a pathway that may contribute to the BthTX-II’s antiparasitic effects. However, the reversible nature of its activity indicates that sustained efficacy may require prolonged exposure or optimised dosing regimens.

This study provides novel insights into the potential of BthTX-II as a biotechnological tool for identifying new therapeutic targets in maternal–foetal toxoplasmosis. Additionally, it enhances our understanding of the molecular mechanisms underlying *T. gondii*–host cell interactions, providing a foundation for future research into the development of targeted therapies for this clinically significant parasitic infection.

## Data Availability

The original data presented in this study are included in this article. Further inquiries can be directed to the corresponding authors.

## Abbreviations

PLA_2_, phospholipases A_2_; svPLA_2_s, snake venom phospholipases A_2_; VEGF, Vascular endothelial growth factor; FBS, Foetal bovine serum; RPMI, Roswell Park Memorial Institute; MOI, Multiplicity of infection; S+P, Sulfadiazine and pyrimethamine, MTT, 3-[4,5-dimethylthiazol-2-yl]-2,5 diphenyltetrazolium bromide; LDH, Lactate dehydrogenase; SDS, Sodium dodecyl sulphate, DMF, *N*,*N*-dimethylformamide; I, Interleukin; TNF-α, Tumour necrosis factor-alpha; MIF, Macrophage migration inhibitory factor; ELISA, Enzyme-linked immunosorbent assay; SEM, standard error of the mean; IC50, half-maximal inhibitory concentration; VEGFR-2, vascular endothelial growth factor receptor 2; ERK1/2, Extracellular signal-regulated kinase 1/2; HE, Haematoxylin–eosin.
